# Pancreatic cancer-derived exosomes promote tumor metastasis and liver pre-metastatic niche formation

**DOI:** 10.18632/oncotarget.18831

**Published:** 2017-06-28

**Authors:** Zeqian Yu, Susu Zhao, Long Ren, Lishan Wang, Zhangjun Chen, Robert M. Hoffman, Jiahua Zhou

**Affiliations:** ^1^ Department of Hepatic-Biliary-Pancreatic Center, Zhongda Hospital, Southeast University, Nanjing, China; ^2^ Department of Hepatobiliary Surgery Research Institute, Southeast University, Nanjing, China; ^3^ Department of Pathology, Traditional Chinese Medicine Hospital of Jiangsu Province, Nanjing, China; ^4^ Department of Surgery, University of California at San Diego, San Diego, California, USA; ^5^ AntiCancer, Inc., San Diego, California, USA

**Keywords:** pancreatic cancer, exosomes, pre-metastatic niche, proteomics, iTRAQ

## Abstract

Exosomes play important roles in cell-cell communication, and are likely mediators of the metastatic cascade in cancer. This study examined the role of exosomes in pancreatic cancer cell adhesion, migration, and invasion. We isolated and purified exosomes from two isogenic pancreatic cancer cell lines with different metastatic potentials. Uptake of exosomes from highly metastatic Panc02-H7 cells decreased adhesion and increased migration and invasion capacity in weakly metastatic Panc02 cells *in vitro*. Exosomes from highly metastatic pancreatic cancer cells induced liver pre-metastatic niche formation in naïve mice and promoted primary tumor growth and liver metastasis *in vivo*. We identified 4,517 proteins in exosomes from Panc02 and Panc02-H7 cells via iTRAQ quantitative proteomic analyses, 79 of which were differentially expressed between the two cell lines. Bioinformatics analyses showed that most of the differentially expressed proteins were involved in pancreatic cancer growth, invasion, and metastasis, and that metabolism-related signaling pathways were involved in exosome-mediated intracellular communication. Further studies will be needed to determine whether these proteins are potential pancreatic cancer diagnostic/prognostic markers or novel therapeutic targets.

## INTRODUCTION

Pancreatic ductal adenocarcinoma (PDAC) is one of the most aggressive human malignancies, and is one of the five most common causes of cancer mortality world wide. An estimated 53,070 new pancreatic cancer cases will be diagnosed and 41,7800 patients will die from pancreatic cancer in the United States in 2017 [1]. Rising incidence and mortality rates were also observed in 2015 in China [2]. Despite some progress in surgical techniques, chemotherapy, and radiotherapy, pancreatic cancer patient prognosis is extremely poor, with an overall 5-year survival rate <6%. Median survival is 8–12 months for patients with locally advanced disease, and only 3–6 months for those with metastatic disease [3]. Accurate diagnostic biomarkers to detect pancreatic cancer at an earlier stage, and novel therapeutic strategies that target metastatic disease are urgently needed to improve patient outcomes.

The tumor microenvironment comprises cancer and stromal cells, along with extracellular matrix (ECM) components and secreted soluble factors, such as chemokines, cytokines, and growth factors. These soluble factors facilitate cell-cell communication, tumor progression and metastasis, and pre-metastatic niche generation in distant organs. Recent studies identified tumor-derived exosomes as key modulators of the tumor microenvironment that mediate cell-cell communication and eventually induce metastatic niche formation [4].

Exosomes are 40–100 nm diameter vesicles derived from late endosome/multivesicular body (MVB) luminal membranes, and are constitutively released via fusion of MVBs with the cell membrane under both physiological and pathological conditions. Exosomes are enriched in parent cell-derived bioactive molecules, including proteins, RNAs, and lipids, which can be horizontally transferred to recipient cells and regulate their function [5]. Specific proteins have been identified in PDAC exosomes [6–10], but a comparative proteomics analysis of exosomes secreted by PDAC cell lines of different metastatic potential has not been performed. The present study used iTRAQ-quantitative proteomic analysis to identify the protein compositions of exosomes derived from two isogenic PDAC cell lines, Panc02 and Panc02-H7, which differ in their degree of metastatic potential [11]. We identified potential key proteins that facilitate crosstalk between the primary tumor and the microenvironment, and which may be useful biomarkers for PDAC diagnosis and prognosis.

## RESULTS

### Panc02 EXO and Panc02-H7 EXO characterization

**Figure 1:**
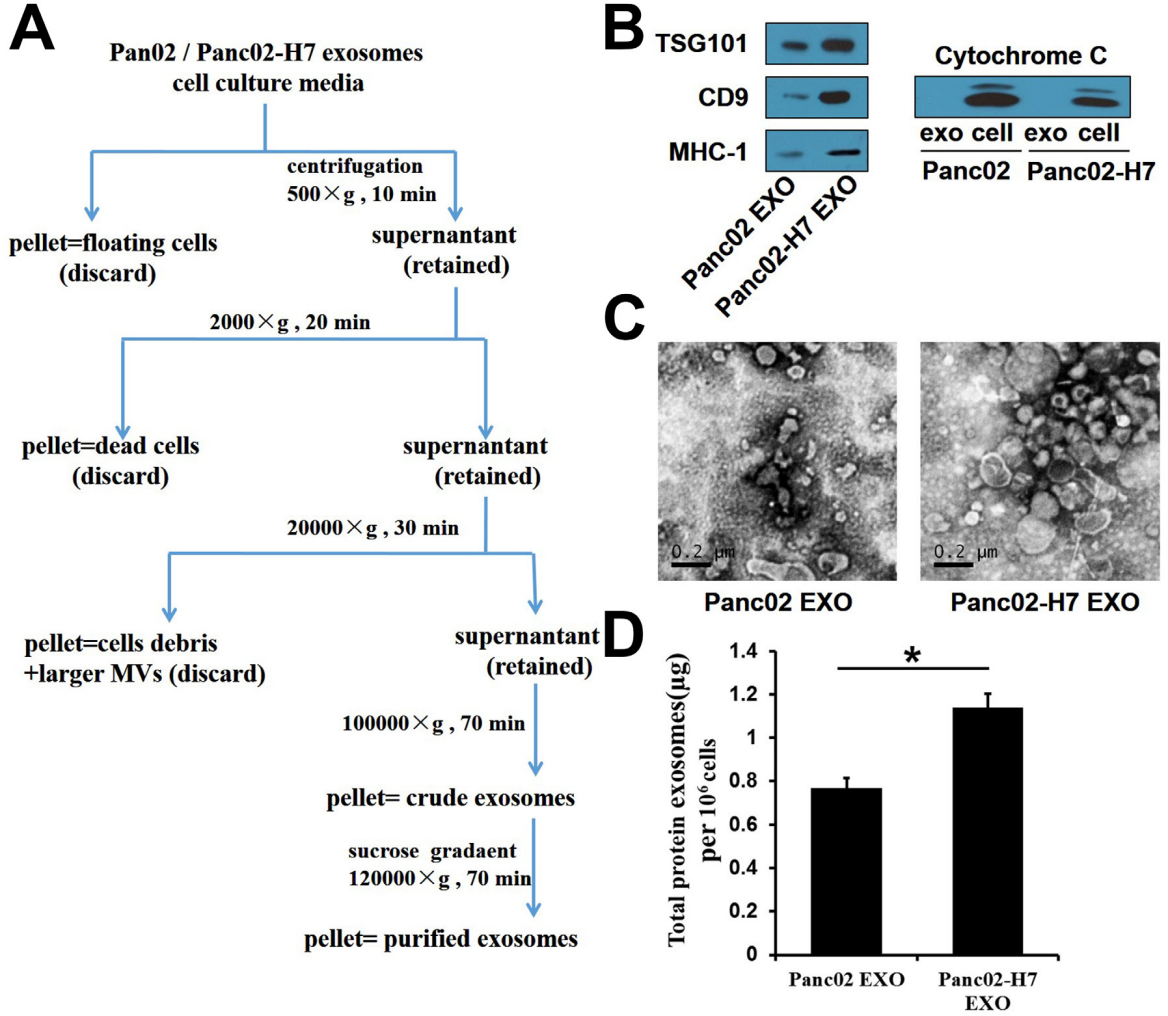
Characterization of Panc02- and Panc02-H7-derived exosomes Exosome isolation and purification schematic. **(A)** Common exosome markers, including TSG101, CD9, and MHC-I, were detected in two exosomes. **(B)** Cytochrome c was detectable in two whole-cell lysates, but not in exosomes. Panc02 EXOs and Panc02-H7 EXOs were negatively stained with 3%phosphotungstic acid and viewed by TEM (scale bar=200 nm). **(C)** Total protein per million cells in two exosomes. **(D)** Panc02-H7EXOs expressed more total protein than Panc02 EXOs.(*P<0.05).

Purified exosomes were isolated using ultracentrifugation combined with sucrose density gradient centrifugation (Figure 1A). Western blotting confirmed the presence of several classical common exosome markers, including TSG101, CD9, and MHC-I (Figure 1B). Cytochrome c, a mitochondrial marker, was detected in two whole cell lysates, but was absent from exosomes, indicating that the exosomes were not contaminated with other vesicles (Figure 1B). Sizes and morphological characteristics of exosomes derived from two pancreatic cancer cell lines were assessed via transmission electron microscopy (TEM). Exosomes from both cell lines were cup-shaped and ranged from 50–150 nm in diameter (Figure 1C). Exosomes from the highly metastatic pancreatic cancer cell line (Panc02-H7 EXO) expressed more total protein than exosomes from the poorly metastatic cell line (Panc02 EXO) (Figure 1D).

### Panc02-H7-derived exosomes decreased adhesion and increased migration and invasion in recipient cells

**Figure 2:**
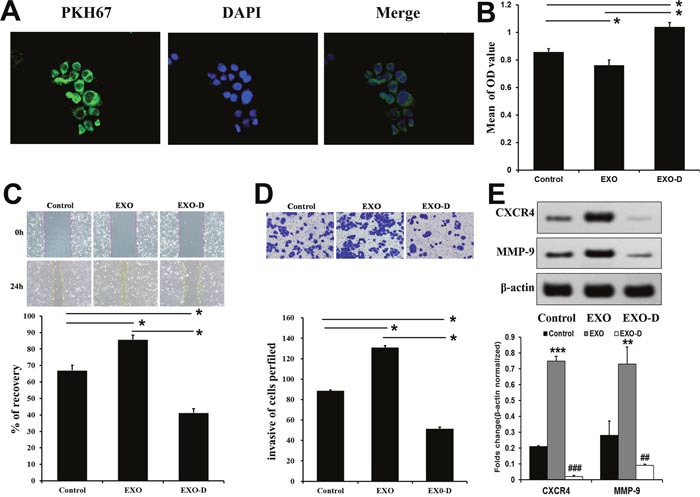
Panc02-H7-derived exosomes promote metastatis-related characteristics *in vitro* Panc02 cells tookup PKH67-labeled Panc02-H7EXOs. Numerous green fluorescently-labeled exosomes were observed inside cells after 5 h (400× magnification). **(A)** The MTT cell adhesion assay indicated that Panc02-H7 EXOs decrease Panc02 cell adhesion. **(B)** Wound-healing assays indicated that Panc02-H7 EXOs enhanced Panc02 cell migration (200×magnification). **(C)** Transwell chamber invasion assays showed that Panc02-H7 EXOs increased Panc02 cell invasion (200×magnification). **(D)** Western blotting indicated that Panc02-H7 EXOs increased Panc02 cell migration and invasion via CXCR4 and MMP-9 signaling. **(E)** n=3/group.*P<0.05,**P<0.01, ***P<0.001 compared to control; #P<0.05, ##P<0.01, ###P<0.001 compared to EXO-D.

We incubated PKH67-labeled Panc02-H7 EXOs with Panc02 cells. After five h, numerous green fluorescent exosomes were observed inside Panc02 cells via fluorescence microscopy. Exosomes were mainly located at the perinuclear region, suggesting Panc02 cell uptake of Panc02-H7 EXOs (Figure 2A). We speculated that exosome cargo release might affect recipient cell metastatic capabilities. Therefore, we analyzed recipient cell adhesive, migratory, and invasive potential following exosome internalization. Amiloride reportedly depresses cell exosome secretion [12]. In the MTT cell adhesion assay, Panc02 cells exposed to PBS (control group), 100 μg/ml Panc02-H7 EXOs (EXO group), or 7 mmol/Lamiloride (Exo-D group) for 24 h were inoculated into 96-well plates in serum-free medium. Cell attachment was greater in the Exo-D group than in the EXO and control groups (Figure 2B), indicating that Panc02-H7 EXOs may decrease Panc02 cell adhesion. In a wound-healing assay, migration was increased in the EXO group compared to the control and Exo-D groups (Figure 2C). Similarly, transwell chamber invasion assays results showed increased invasion by EXO group cells compared to control and Exo-D cells (Figure 2D). Western blotting results showed that CXCR4 and MMP-9 levels were higher in the EXO group than in the control group, but were reduced in the Exo-D group (Figure 2E). These results suggest that exosomes from high metastatic-potential cells can induce metastatic behavior in Panc02 cells.

### Exosome tissue distribution and liver pre-metastatic niche formation

**Figure 3:**
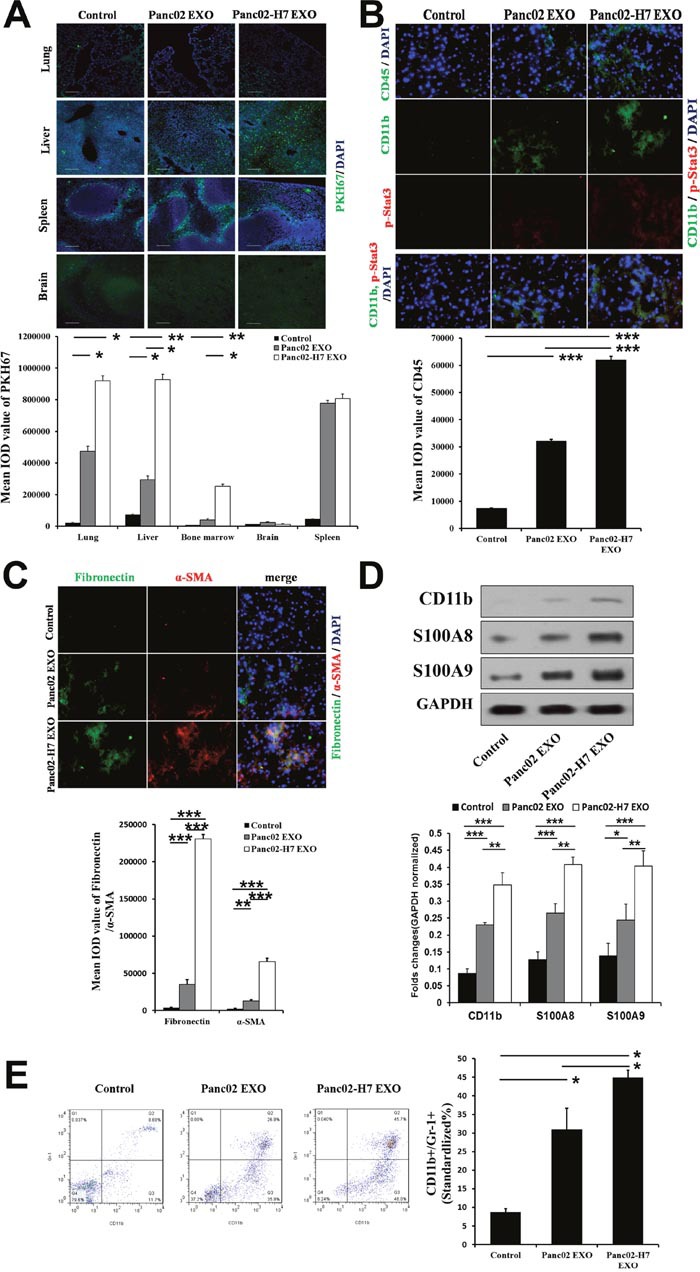
Pancreatic cancer-derived exosomes mediate liver pre-metastatic niche formation Confocal microscopy (lung, liver, spleen, brain) of PKH67-labeled Panc02 EXO and Panc02-H7 EXO tissue distribution (green) 24 hpi. **(A)** PKH-67-labeled liposomes served as controls (scale bar=100 μm). Histogram shows exosome tissue distribution quantification (n=5/group). CD45, p-Stat3, and CD11b IF staining in liver sections from controls (left) and mice treated with Panc02 EXOs(middle) or Panc02-H7 EXOs (right) for 12 d without tumor challenge. **(B)** Histogram shows infiltrating CD45^+^ cell quantification. FN and α-SMA IF staining in liver sections from controls (top) and mice treated with Panc02 EXOs(middle) or Panc02-H7 EXOs (bottom) for 12 d without tumor challenge. **(C)** Histogram shows infiltrating α-SMA^+^hStCs and FN expression quantification(400× magnification; n=5/group). Western blotting analysis showed upregulated S100A8 and S100A9 in livers treated with Panc02-H7-derived exosomes. Histogram shows expression of the three proteins in three groupsas determined by densitometric analysis (n=3/group). **(D)** Pancreatic cancer-derived exosomes induce MDSC accumulation in peripheral blood. **(E)** Representative flow cytometric plots (left) and quantification (right) of CD11b^+^GR1^+^ MDSCs (n=5/group). *P<0.05, **P<0.01,***P<0.001.

Exosome biodistribution in liver, lung, spleen, kidney, brain, and bone marrow was assessed 24h post-injection (hpi) of exosomes using confocal microscopic analysis. Pancreatic cancer-derived exosomes accumulated in the lung, liver, and spleen, with less accumulation in the brain and bone marrow compared to a liposome control (Figure 3A). Panc02-H7 cell-derived exosomes accumulated at higher levels in the lung, liver, and bone marrow than exosomes from Panc02 cells (Figure 3A).

Immunofluorescence (IF) quantitative analysis showed that Panc02-H7 EXOs increased the frequency of CD11b^+^ (also confirmed by Western blotting) and CD45^+^ hematopoietic progenitor cells at pre-metastatic sites after 12 d compared with Panc02 EXO and control groups (Figure 3B). Pancreatic cancer-derived exosomes also induced Stat3 activation and myeloid infiltration (over 12 d of treatment). Stat3 activity was detectable in myeloid cells and in the liver (Figure 3B). We observed increased alpha smooth muscle actin (α-SMA)^+^ hepatic stellate cells (hStCs) and fibronectin (FN) upregulation in mice treated with Panc02-H7 EXOs compared with Panc02 EXOs and controls. We found a predominant α-SMA^+^ cell population in FN-enriched liver areas in mice treated with Panc02-H7 EXOs, suggesting that activated hStCs produced most of the FN (Figure 3C). Western blotting analysis showed upregulated S100A8 and S100A9 in Panc02-H7 EXO-treated livers (Figure 3D). Panc02-H7 EXOs increased myeloid-derived suppressor cell (MDSC) (CD11b^+^GR1^+^cell) frequency in peripheral blood more than Panc02 EXOs and the control group (Figure 3E).

### Tumor-derived exosomes promote pancreatic cancer growth, micrometastasis, and metastasis

**Figure 4:**
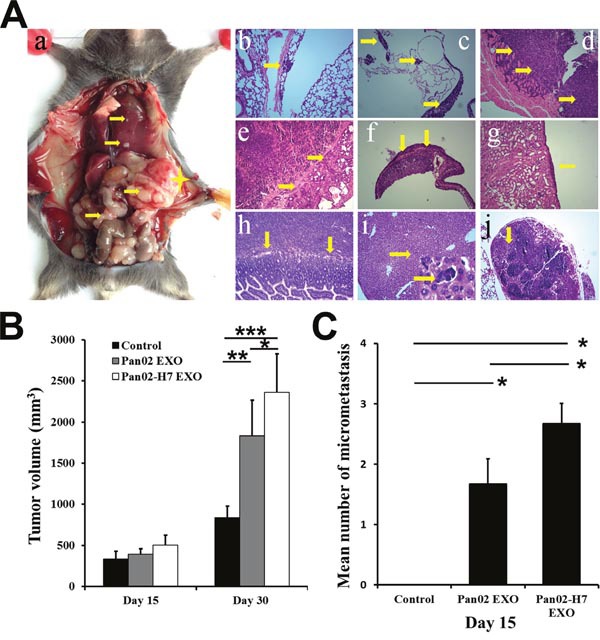
Pancreatic cancer-derived exosomes promote tumor growth and metastasis Representative macro-anatomy and H&E-stained images of Panc02-H7 EXO-treated mice 30 d post-SOI. **(A)** A yellow star marks primary tumor locations; arrows indicate metastatic sites in the peritoneal cavity **(Aa)**. Arrows showing lung micrometastasis **(Ab)**, pleura metastasis **(Ac)**, tumor invasion of the diaphragm **(Ad)**, metastasis to spleen **(Ae)**, tumor invasion of the adrenal gland **(Af)**, metastasis to the kidney **(Ag)**, tumor invasion of the small intestine **(Ah)**, micrometastasis of the liver(lower right shows enlarged image) **(Ai)**, and metastasis to lymph nodes **(Aj)** (magnification, 100×). Primary tumor volume in mice treated with PBS, Panc02 EXOs, orPanc02-H7 EXOs at 15 and 30 d post-SOI **(B)**. Number of micrometastases in mice treated with PBS, Panc02 EXOs, or Panc02-H7 EXOsat 15d post-SOI (n=6/group) **(C)**. *P<0.05, **P<0.01,***P<0.001.

We administed Panc02-H7 EXOs and Panc02 EXOs (10 μg) intravenously (tail vein) into C57B/L6 mice three d per week, starting seven d after pancreatic cancer surgical orthotopic implantation (SOI). Primary tumor volume only increased at 30 d post-SOI (Figure 4B). In contrast to controls, only Panc02-H7 EXO-treated mice exhibited lung and liver micrometastasis at d 15 (Figure 4C). These mice also had a greater metastatic burden and cancer cell distribution in the liver, lung, diaphragm, pleura, adrenal gland, small intestine, lymph nodes, and spleen compared to PBS- or Panc02 EXO-treated mice (Figure 4A). These data suggest that qualitative exosome content differences can mediate metastatic potential and organ otropism.

**Figure 5:**
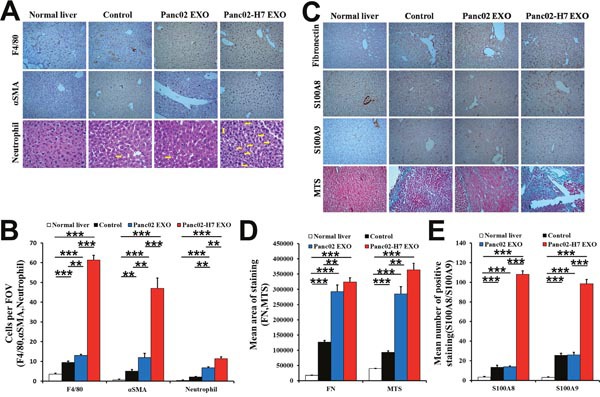
Pancreatic cancer-derived exosomes induce inflammation and fibrotic microenvironment formation in the liver IHC analysis and histopathological examination of macrophages (F4/80), hStCs (α-SMA), and neutrophils in liver metastatic niches of naïve mice and mice treated with PBS, Panc02 EXOs, or Panc02-H7 EXOs at 30d post-SOI (arrow shows neutrophils in liver) **(A)**. Representative histogram shows quantification of F4/80^+^ macrophages, α-SMA^+^ hStCs, and neutrophils **(B)**. Identification of FN, S100A8, and S100A9 as inflammatory mediators, and collagen deposition in the liver metastatic niche **(C)**. Representative histogram shows FN and MTS quantification **(D)**. Representative histogram shows S100A8 and S100A9 quantification **(E)**. n=6/group.**P<0.01,***P<0.001.10 fields assessed per sample. FOV, field of view.

S100A8 and S100A9 were upregulated, and F4/80^+^ macrophages, α-SMA^+^hStCs, and neutrophils were increased in Panc02-H7 EXO-treated mouse livers compared with untreated, Panc02 EXO-, or PBS-treated livers (Figure 5A–5B & 5C–5E). There was no difference between the Panc02 EXO and control groups. Immunohistochemical (IHC) analyses and Masson's trichrome staining showed that FN was upregulated in Panc02-H7 EXO-treated mouse livers. Connective tissue deposition was increased in mouse livers treated with either type of exosome compared with control and normal liver, with no difference between the two exosomes (Figure 5C–5D). These data suggest that pancreatic cancer-derived exosomes may reprogram the liver to form liver metastatic niches.

### Exosomal protein identification via iTRAQ-based proteomic analysis

**Figure 6:**
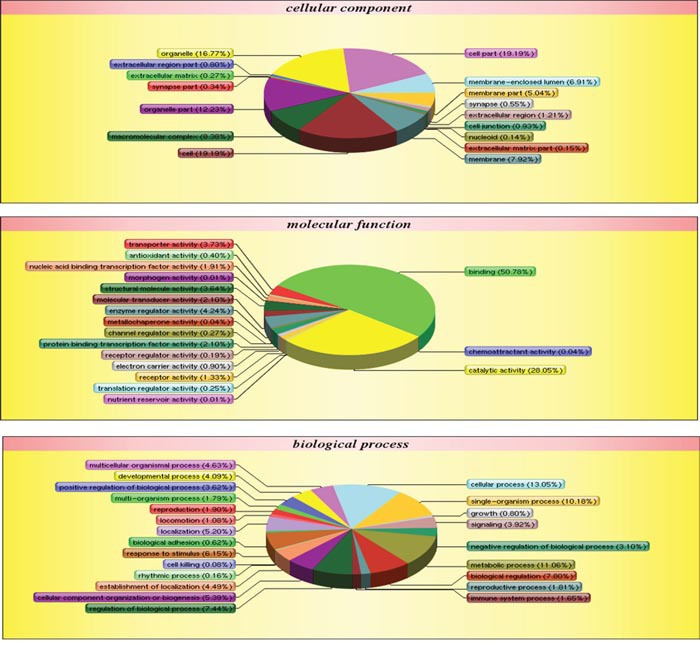
Panc02 EXO and Panc02-H7 EXO proteins identified via iTRAQ quantitative proteomic analysis GO enrichment analysis of 4,517exosomal proteins via Blast2go. Proteins were classified by cellular component (CC), molecular function (MF), or biological process (BP).

Ultimately, 65,077 unique spectra, 22,912 unique peptides, and 4,517 proteins were identified via iTRAQ-based proteomic analysis in the two types of pancreatic cancer exosomes, 71.09% of which were identified with ≥2 peptide matches (Supplementary Table 1). The 4,517 common exosome proteins were classified into three groups, cellular components, molecular function, and biological process, through Blast2go to assess gene ontology (GO) enrichment (Figure 6). Based on different molecular functions, these proteins were grouped as follows: binding, catalytic activity, enzyme regulator activity, transporter activity, structural molecule activity, and transcription factors. The common exosomal proteins are mainly involved in metabolic, structural, and regulating processes. (Table 1) shows the top 25 pathways involving these proteins.

**Table 1:** 79 proteins were differentially-expressed between Panc02 EXOs and Panc02-H7 EXOs

Hits	Accession	Protein name	Expression	114/116	119/118	Mean
1	sp|O88477|IF2B1_MOUSE	Insulin-like growth factor 2 mRNA-binding protein 1	up	1.937	2.205	2.071
2	tr|G3XA48|G3XA48_MOUSE	Isopentenyl-diphosphate Delta-isomerase 1	up	1.548	1.272	1.41
3	sp|P70460|VASP_MOUSE	Vasodilator-stimulated phosphoprotein	up	1.429	1.514	1.472
4	sp|P47930|FOSL2_MOUSE	Fos-related antigen 2	up	1.596	1.687	1.642
5	tr|Q3TT92|Q3TT92_MOUSE	Dihydropyrimidinase-related protein 3	up	3.193	3.973	3.583
6	sp|Q8K2Q9|SHOT1_MOUSE	Shootin-1	up	1.485	1.548	1.517
7	tr|Q3U125|Q3U125_MOUSE	Redox-regulatory protein FAM213A	up	2.076	1.796	1.936
8	tr|Q3TXR4|Q3TXR4_MOUSE	Transcription factor jun-B	up	1.442	1.639	1.541
9	sp|P50543|S10AB_MOUSE	Protein S100-A11	up	2.692	1.942	2.317
10	tr|Q3TSQ1|Q3TSQ1_MOUSE	Sodium/potassium-transporting ATPase subunit beta	up	1.917	1.813	1.865
11	sp|Q8BV49|IFIX_MOUSE	Pyrin and HIN domain-containing protein 1	up	1.548	1.573	1.561
12	sp|P18406|CYR61_MOUSE	Protein CYR61	up	1.359	1.529	1.444
13	sp|Q9CQ69|QCR8_MOUSE	Cytochrome b-c1 complex subunit 8	up	1.353	1.983	1.668
14	sp|P05784|K1C18_MOUSE	Keratin, type I cytoskeletal 18	up	1.555	1.536	1.546
15	sp|Q9CR98|F136A_MOUSE	Protein FAM136A	up	1.539	1.254	1.397
16	tr|E0CXM9|E0CXM9_MOUSE	Zinc finger protein-like 1	up	1.68	1.05	1.365
17	sp|Q9R1Q7|PLP2_MOUSE	Proteolipid protein 2	up	2.792	1.279	2.036
18	sp|Q91XV3|BASP1_MOUSE	Brain acid soluble protein 1	up	2.963	3.513	3.238
19	sp|Q9DCV7|K2C7_MOUSE	Keratin, type II cytoskeletal 7	up	4.188	4.47	4.329
20	tr|F6ZFU0|F6ZFU0_MOUSE	Elongation factor 1-delta	up	1.016	1.578	1.297
21	sp|Q8R5J9|PRAF3_MOUSE	PRA1 family protein 3	up	1.32	1.599	1.46
22	tr|D3YUW7|D3YUW7_MOUSE	Cingulin	up	2.034	2.236	2.135
23	tr|Q3U7D2|Q3U7D2_MOUSE	Ribosomal protein L15	up	1.52	1.049	1.285
24	tr|Q3TGQ3|Q3TGQ3_MOUSE	Putative uncharacterized protein	up	1.401	1.53	1.466
25	tr|Q3TLJ9|Q3TLJ9_MOUSE	Putative uncharacterized protein	up	1.553	1.357	1.455
26	sp|P62077|TIM8B_MOUSE	Mitochondrial import inner membrane translocase subunit Tim8	up	1.51	1.359	1.435
27	tr|G5E850|G5E850_MOUSE	Cytochrome b-5, isoform CRA_a	up	1.323	1.538	1.431
28	sp|Q8BGZ7|K2C75_MOUSE	Keratin, type II cytoskeletal 75	up	1.203	1.771	1.487
29	tr|D3Z125|D3Z125_MOUSE	Tumor protein D52	up	1.596	1.385	1.491
30	sp|P45377|ALD2_MOUSE	Aldose reductase-related protein 2	up	1.759	1.442	1.601
31	tr|B2RUC1|B2RUC1_MOUSE	Tpd52l1 protein	up	1.425	1.699	1.562
32	sp|P19001|K1C19_MOUSE	Keratin, type I cytoskeletal 19	up	1.727	1.819	1.773
33	tr|A2A547|A2A547_MOUSE	Ribosomal protein L19	up	1.166	1.502	1.334
1	tr|Q9D089|Q9D089_MOUSE	Putative uncharacterized protein	down	0.929	0.597	0.763
2	tr|J3QN31|J3QN31_MOUSE	Adenylosuccinate synthetase isozyme 1	down	0.499	0.504	0.502
3	tr|F8VQJ3|F8VQJ3_MOUSE	Laminin subunit gamma-1	down	0.57	0.644	0.607
4	sp|Q3UHD3|MTUS2_MOUSE	Microtubule-associated tumor suppressor candidate 2 homolog	down	0.852	0.646	0.749
5	sp|Q61599|GDIR2_MOUSE	Rho GDP-dissociation inhibitor 2	down	0.731	0.588	0.66
6	tr|Q3TDU5|Q3TDU5_MOUSE	Milk fat globule-EGF factor 8 protein, isoform CRA_a	down	0.688	0.645	0.667
7	sp|P16125|LDHB_MOUSE	L-lactate dehydrogenase B chain	down	0.179	0.165	0.172
8	sp|Q8R3P0|ACY2_MOUSE	Aspartoacylase	down	0.549	0.619	0.584
9	sp|Q6ZPE2|MTMR5_MOUSE	Myotubularin-related protein 5	down	0.634	0.875	0.755
10	sp|Q8BFU3|RN214_MOUSE	RING finger protein 214	down	0.652	0.866	0.759
11	sp|Q61553|FSCN1_MOUSE	Fascin	down	0.273	0.282	0.278
12	sp|P21981|TGM2_MOUSE	Protein-glutamine gamma-glutamyltransferase 2	down	0.711	0.605	0.658
13	sp|P17563|SBP1_MOUSE	Selenium-binding protein 1	down	0.488	0.528	0.508
14	sp|Q810Q5|NMES1_MOUSE	Normal mucosa of esophagus-specific gene 1 protein	down	0.807	0.515	0.661
15	tr|E9PVM7|E9PVM7_MOUSE	Glutathione S-transferase Mu 5	down	0.803	0.644	0.724
16	sp|Q8BVA5|CB043_MOUSE	UPF0554 protein C2orf43 homolog	down	0.536	0.358	0.447
17	tr|A2AE89|A2AE89_MOUSE	Glutathione S-transferase Mu 1	down	0.473	0.652	0.563
18	sp|P48428|TBCA_MOUSE	Tubulin-specific chaperone A	down	0.661	0.76	0.711
19	sp|Q4VAA2|CDV3_MOUSE	Protein CDV3	down	0.556	0.91	0.733
20	sp|P06801|MAOX_MOUSE	NADP-dependent malic enzyme	down	0.712	0.661	0.687
21	sp|P60824|CIRBP_MOUSE	Cold-inducible RNA-binding protein	down	0.671	0.62	0.646
22	tr|Q3UGY5|Q3UGY5_MOUSE	Putative uncharacterized protein	down	0.441	0.523	0.482
23	sp|Q8BMK4|CKAP4_MOUSE	Cytoskeleton-associated protein 4	down	0.336	0.529	0.433
24	sp|P05063|ALDOC_MOUSE	Fructose-bisphosphate aldolase C	down	0.651	0.558	0.605
25	tr|E0CY47|E0CY47_MOUSE	1-phosphatidylinositol 4,5-bisphosphate phosphodiesterase eta-1	down	0.649	0.628	0.639
26	tr|Q3TNC8|Q3TNC8_MOUSE	Putative uncharacterized protein	down	0.632	0.522	0.577
27	sp|Q60928|GGT1_MOUSE	Gamma-glutamyltranspeptidase 1	down	0.589	0.592	0.591
28	sp|P14873|MAP1B_MOUSE	Microtubule-associated protein	down	0.62	0.618	0.619
29	tr|Q5F2B1|Q5F2B1_MOUSE	Mannose-P-dolichol utilization defect 1 protein	down	0.797	0.661	0.729
30	tr|Q3TJK3|Q3TJK3_MOUSE	Putative uncharacterized protein	down	0.551	0.468	0.51
31	tr|Q3TDX7|Q3TDX7_MOUSE	Extracellular matrix protein 1	down	0.451	0.388	0.42
32	tr|Q8CEU1|Q8CEU1_MOUSE	Putative uncharacterized protein	down	0.644	0.74	0.692
33	sp|P02798|MT2_MOUSE	Metallothionein-2	down	0.727	0.65	0.689
34	tr|I1E4×1|I1E4×1_MOUSE	Syntaxin-5	down	0.642	0.59	0.616
35	tr|Q3UNF3|Q3UNF3_MOUSE	Acyl-coenzyme A oxidase	down	0.726	0.646	0.686
36	tr|Q4VAF0|Q4VAF0_MOUSE	Acylphosphatase	down	0.87	0.613	0.742
37	tr|Q3TXK6|Q3TXK6_MOUSE	Putative uncharacterized protein	down	0.716	0.606	0.661
38	tr|G3×8Q5|G3×8Q5_MOUSE	Ceruloplasmin	down	0.741	0.555	0.648
39	tr|K4DI77|K4DI77_MOUSE	WD repeat-containing protein 81	down	0.663	0.885	0.774
40	tr|Q3TVJ0|Q3TVJ0_MOUSE	Putative uncharacterized protein	down	0.563	0.359	0.461
41	tr|Q1KYM0|Q1KYM0_MOUSE	Env polyprotein	down	0.661	0.614	0.638
42	tr|Q3THB4|Q3THB4_MOUSE	L-lactate dehydrogenase	down	0.565	0.348	0.457
43	tr|Q3U3U6|Q3U3U6_MOUSE	Putative uncharacterized protein	down	0.631	0.97	0.801
44	sp|Q91ZX7|LRP1_MOUSE	Pro-low-density lipoprotein receptor-related protein 1	down	0.577	0.708	0.643
45	tr|Q3U2K1|Q3U2K1_MOUSE	Putative uncharacterized protein	down	0.629	0.915	0.772
46	sp|Q8R180|ERO1A_MOUSE	ERO1-like protein alpha	down	0.674	0.602	0.638

33 proteins were upregulated and 46 were downregulated. Fold changes in abundance were also observed in two additional independent biological replicates.

### Bioinformatic analysis of proteins differentially expressed between high- and low-metastatic exosomes

**Figure 7:**
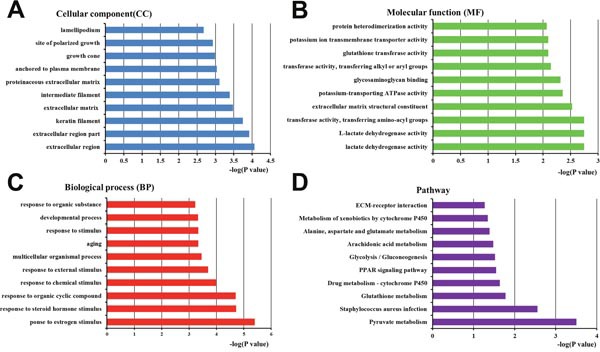
Bioinformatics analysis of proteins differentially expressed between Panc02 EXOs and Panc02-H7 EXOs GO annotation of the final selected differentially expressed proteins, and KEGG pathway analysis. Differentially expressed proteins were classified by cellular component (CC) **(A)**, molecular function (MF) **(B)**, biological process (BP) **(C)**, and pathway analysis **(D)**. The top 10 CC, MF, BP, and pathway analysis components of the selected differentially expressed proteins are shown. Enrichment scores are represented as -log(p-values).

79 proteins were differentially expressed between high- and low-metastatic exosomes (Table 2). We again performed a Blast2go analysis to assess protein gene ontology (GO) enrichment (Figure 7).

**Table 2:** Top 25 KEGG pathways involving common exosome proteins

No.	Pathway	Proteins with pathway annotation	Pathway ID
1	Metabolic pathways	578 (15.72%)	ko01100
2	RNA transport	145 (3.94%)	ko03013
3	Spliceosome	141 (3.84%)	ko03040
4	Pathways in cancer	127 (3.45%)	ko05200
5	Huntington's disease	126 (3.43%)	ko05016
6	Endocytosis	124 (3.37%)	ko04144
7	Protein processing in endoplasmic reticulum	122 (3.32%)	ko04141
8	Epstein-Barr virus infection	122 (3.32%)	ko05169
9	Regulation of actin cytoskeleton	117 (3.18%)	ko04810
10	Alzheimer's disease	113 (3.07%)	ko05010
11	Focal adhesion	106 (2.88%)	ko04510
12	MAPK signaling pathway	96 (2.61%)	ko04010
13	Tight junction	96 (2.61%)	ko04530
14	Parkinson's disease	95 (2.58%)	ko05012
15	HTLV-I infection	94 (2.56%)	ko05166
16	Influenza A	90 (2.45%)	ko05164
17	Purine metabolism	89 (2.42%)	ko00230
18	Oxidative phosphorylation	88 (2.39%)	ko00190
19	Ribosome	86 (2.34%)	ko03010
20	Insulin signaling pathway	82 (2.23%)	ko04910
21	Ubiquitin mediated proteolysis	80 (2.18%)	ko04120
22	mRNA surveillance pathway	79 (2.15%)	ko03015
23	Lysosome	75 (2.04%)	ko04142
24	Herpes simplex infection	72 (1.96%)	ko05168
25	Ribosome biogenesis in eukaryotes	70 (1.9%)	ko03008

### Validation of differentially expressed proteins identified by iTRAQ quantitative proteomics

**Figure 8:**
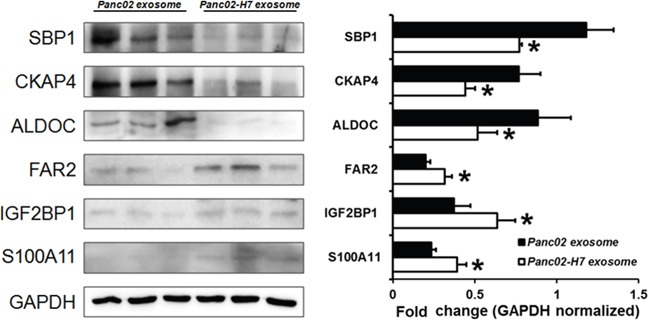
Validation of iTRAQ quantitative proteomic analysis results Western blotting validated differential expression patterns for six candidate proteins. FAR2, IGF2BP1, and S100A11 were upregulated, and SBP1, CKAP4, and ALDOC were down regulated in Panc02-H7 EXOs compared to Panc02 EXOs, in agreement with iTRAQ results.n=3/group. *P<0.05.

We validated six candidate proteins using Western blotting. SBP1, CKAP4, and ALDOC were downregulated, and FAR2, IGF2BP1, and S100A11 were upregulated in Panc02-H7 EXOs compared to Panc02EXOs (Figure 8). GAPDH was used as loading control. Protein band densities analyzed quantitatively using ImageJ verified that expression patterns were consistent with iTRAQ quantitative proteomics results.

## DISCUSSION

There is currently no standardized exosome isolation technique. In this study, we isolated exosomes by collecting cell-conditioned medium from ordinary culture flasks. We purified exosomes using ultracentrifugation combined with sucrose density gradient centrifugation. However, this method was time-consuming, labor intensive and resulted in low yield. Jeppesen, et al. developed an exosome collection strategy using CLAD1000 bioreactors and a 10-kDa semi-permeable membrane. Their method increases exosome yields 13–16-fold compared to isolation from flasks [13].

Exosomes as cell-free messengers play important roles in cell-cell communication, and are likely mediators of the metastatic cascade [5, 14–16]. This study examined the role of exosomes in cancer adhesion, migration, and invasion. Chemokines and their receptors contribute to cancer metastasis, particularly stromal cell-derived factor-1α and its receptor, CXCR4 [17–18]. MMP-9 is a downstream signaling molecule of CXCR4 and is critical for cancer cell migration and invasion [19]. We found that Panc02-H7 EXO treatment increased Panc02 cell migration and invasion, and upregulated CXCR4 and MMP-9 compared with controls, indicating that CXCR4 and MMP-9 signaling may enhance Panc02 cell metastatic capabilities.

Fluorescent labeling allowed for direct exosome visualization in our study. Consistent with other groups, we found that exosome tissue distribution mimicked the organotropic distribution of the cell line of origin [20]. The organotropic nature of exosomes is largely attributed to a specific exosomal surface integrin repertoire that dictates exosome homing to distant organs and uptake by target cells. Our exosome proteomic analysis identified multiple integrin subunits (beta-1, alpha-3, beta-3, beta-4, alpha 5, alpha-V, alpha-6, and alpha-X) (Table 3) that could be associated with pancreatic cancer metastasis to multiple organs. Our model allowed us to study the effects of exosome accumulation in pre-metastatic organs and to determine whether or not such accumulation could prime the liver microenvironment for metastatic tumor cell colonization.

**Table 3:** Selected proteins identified in Panc02 EXOs and Panc02-H7 EXOs via iTRAQ quantitative proteomic analysis

Category	Accession	Protein name	ExoCarta
ESCRT-1	tr|Q3UK08|Q3UK08_MOUSE	TSG 101	Yes
ESCRT-associated	sp|Q8R1T1|CHMP7_MOUSE	CHMP7	Yes
	sp|Q9CQ10|CHMP3_MOUSE	CHMP3	Yes
	tr|Q3TDX2|Q3TDX2_MOUSE	VPS4A	Yes
	sp|Q9WU78|PDC6I_MOUSE	PCD6IP /Alix	Yes
Tetraspanins	sp|Q9CQ88|TSN31_MOUSE	Tetraspanin-31	No
	tr|Q3UC80|Q3UC80_MOUSE	CD63 antigen	Yes
	tr|Q921J7|Q921J7_MOUSE	Tetraspanin/CD151	Yes
Tetraspanins-associated	tr|G3UYZ1|G3UYZ1_MOUSE	IGSFM8	Yes
GTPase	tr|Q5SW88|Q5SW88_MOUSE	RAB1A	Yes
	sp|Q9D1G1|RAB1B_MOUSE	RAB1B	Yes
	sp|P53994|RAB2A_MOUSE	RAB2A	Yes
	tr|Q6P7T7|Q6P7T7_MOUSE	RAB2B	Yes
	tr|B2RRN5|B2RRN5_MOUSE	RAB4A	Yes
	sp|Q9CQD1|RAB5A_MOUSE	RAB5A	Yes
	tr|Q0PD56|Q0PD56_MOUSE	RAB5B	Yes
	tr|Q8C266|Q8C266_MOUSE	RAB5C	Yes
	tr|Q3U4W5|Q3U4W5_MOUSE	RAB6	Yes
	sp|P51150|RAB7A_MOUSE	RAB7A	Yes
	tr|Q3UHW5|Q3UHW5_MOUSE	RAB8A	Yes
	sp|P61028|RAB8B_MOUSE	RAB8B	Yes
	tr|A2AFP4|A2AFP4_MOUSE	RAB9A	Yes
	sp|P61027|RAB10_MOUSE	RAB10	Yes
	sp|P46638|RB11B_MOUSE	RAB11B	Yes
	tr|A2CG35|A2CG35_MOUSE	RAB12	Yes
	tr|Q50HX0|Q50HX0_MOUSE	RAB14	Yes
	sp|P35293|RAB18_MOUSE	RAB18	Yes
	tr|Q6A0C7|Q6A0C7_MOUSE	RAB21	Yes
	tr|A2ARZ7|A2ARZ7_MOUSE	RAB22A	Yes
	tr|Q3TXV4|Q3TXV4_MOUSE	RAB22B	Yes
	tr|Q9D4I9|Q9D4I9_MOUSE	RAB23	Yes
	tr|Q0PD20|Q0PD20_MOUSE	RAB34	Yes
	sp|Q6PHN9|RAB35_MOUSE	RAB35	Yes
	tr|Q3UZM8|Q3UZM8_MOUSE	RAB40C	No
Syntenin	sp|Q99JZ0|SDCB2_MOUSE	Syntenin-2	Yes
SNARE	tr|B0QZN5|B0QZN5_MOUSE	VSMP 2	Yes
	sp|P63024|VAMP3_MOUSE	VSMP 3	Yes
	tr|Q8BSN6|Q8BSN6_MOUSE	VSMP 4	No
	sp|P70280|VAMP7_MOUSE	VSMP 7	Yes
	sp|O70404|VAMP8_MOUSE	VSMP 8	Yes
Internalization motif	tr|A2APM1|A2APM1_MOUSE	CD44 antigen	Yes
	sp|Q62351|TFR1_MOUSE	Transferrin receptor protein 1	Yes
Protein binding domain	sp|Q9WV91|FPRP_MOUSE	Prostaglandin F2 receptor negative regulator	Yes
Heat shock protein	sp|P17879|HS71B_MOUSE	Heat shock 70 kDa protein 1B	Yes
	tr|Q3UIF3|Q3UIF3_MOUSE	Heat shock protein HSP 90-alpha	Yes
	tr|Q3UBU0|Q3UBU0_MOUSE	Hsp90b1	Yes
	sp|Q61699|HS105_MOUSE	Heat shock protein 105 kDa	Yes
Annexin	tr|Q3U5N9|Q3U5N9_MOUSE	Annexin A1	Yes
	tr|Q9CZI7|Q9CZI7_MOUSE	Annexin A2	Yes
	tr|Q3U737|Q3U737_MOUSE	Annexin A3	Yes
	sp|P97429|ANXA4_MOUSE	Annexin A4	Yes
	sp|P48036|ANXA5_MOUSE	Annexin A5	Yes
	tr|Q3TUI1|Q3TUI1_MOUSE	Annexin A6	Yes
	tr|Q3TJ49|Q3TJ49_MOUSE	Annexin A7	Yes
	tr|Q921D0|Q921D0_MOUSE	Annexin A8	Yes
	sp|P97384|ANX11_MOUSE	Annexin A11	Yes
Adhesion	tr|Q3UGY5|Q3UGY5_MOUSE	Fibronectin	Yes
	sp|P09055|ITB1_MOUSE	Integrin beta-1	Yes
	sp|Q62470|ITA3_MOUSE	Integrin alpha-3	Yes
	sp|O54890|ITB3_MOUSE	Integrin beta-3	Yes
	sp|A2A863|ITB4_MOUSE	Integrin beta-4	Yes
	tr|Q80YP5|Q80YP5_MOUSE	Integrin alpha 5	Yes
	tr|Q6PEE8|Q6PEE8_MOUSE	Integrin alpha-6	Yes
	tr|A2AKI5|A2AKI5_MOUSE	Integrin alpha-V	Yes
S100	sp|P07091|S10A4_MOUSE	Protein S100-A4	Yes
	sp|P14069|S10A6_MOUSE	Protein S100-A6	Yes
	tr|Q3UF30|Q3UF30_MOUSE	Protein S100-A10	Yes
	sp|P50543|S10AB_MOUSE	Protein S100-A11	Yes
	sp|P97352|S10AD_MOUSE	Protein S100-A13	Yes
	sp|P50114|S100B_MOUSE	Protein S100-B	Yes

Presence or absence of detected proteins in the ExoCarta database is noted.

The liver is the main site of metastatic disease from gastrointestinal malignancies, such as pancreatic, colon, and gastric carcinomas. The term “liver pre-metastatic niche” was coined to describe a liver micro-environment permissive to metastatic outgrowth in advance of cancer cell arrival, established through soluble-factor activity and exosome release by the primary tumor. The liver metastatic microenvironment is composed of resident Kupffer cells (KCs), hStCs, bone marrow-derived cells (BMDCs), extracellular matrix components, and secreted soluble factors, such as cytokines and chemokines. Costa-Silva, et al. showed that PDAC-derived exosomes containing macrophage migration inhibitory factor (MIF) are selectively taken up by hepatic KCs, upregulating TGF-β [8]. This increases fibronectin production by hStCs and promotes liver recruitment of bone marrow-derived neutrophils and macrophages, completing pre-metastatic niche formation. Nielsen, et al. showed that metastasis-associated macrophages (MAMs) activate resident hStC transformation into myofibroblasts, resulting in a fibrotic microenvironment that sustains metastatic tumor growth [21]. Macrophages in the liver include embryonically derived tissue-resident macrophages (KCs) and infiltrating macrophages derived from inflammatory monocytes (IMs) that originate from the bone marrow (BM) [22]. These macrophages can also trigger hStC activation and fibrogenesis [23], a process important in the early stages of extra-vascular tumor expansion. Both of these studies emphasized that macrophages play key roles in PDAC liver metastasis. Our IHC analysis results also showed that F4/80^+^ macrophages were increased in Panc02-H7 EXO-treated livers compared with controls and normal liver. However, F4/80, a cell surface marker used to identify KCs, is also expressed on recruited monocytes, and strategies used to eliminate macrophages *in vivo* are not KC specific. Thus, our results require further verification.

Under physiological conditions, quiescent hStCs in the space of Disse [24] become activated myofibroblasts that express α-SMA and produce ECM rich in collagens I and IV in response to liver damage, inflammatory stimuli, and tumor cells [25–26]. Chemokines and cytokines released by α-SMA^+^ hStCs also recruit inflammatory/immune cells and enhance premetastatic niche formation. We observed increased α-SMA^+^hStC frequencies in Panc02-H7 EXO-treated livers, suggesting that activated α-SMA^+^ hStCs produced most of the FN in both the pre-metastatic and metastatic niches. Masson's trichrome staining also showed higher degrees of fibrosis in livers treated with either Panc02- or Panc02-H7-derived exosomes. We also found that Panc02-H7 EXO treatment increased neutrophil number and upregulated S100A8 and S100A9 in the mouse liver. Neutrophils may be mobilized into liver premetastatic niches by S100A8 and S100A9 or chemokines and cytokines secreted by activated macrophages, endothelial cells, or cancer cells.

Exosome treatment increased both CD11b^+^ and CD45^+^ hematopoietic progenitor cells in the liver, and activated Stat3 in myeloid cells. Wen, et al. showed that highly metastatic breast cancer-derived exosomes were taken up by CD45^+^BMDCs [27]. Subsequent conditioning of naïve mice promoted MDSC accumulation and immune suppressive microenvironment formation in the lung and liver. Breast cancer exosomes also directly suppressed T-cell proliferation and inhibited NK cell cytotoxicity, likely suppressing the anti-cancer immune response in pre-metastatic organs [27]. Our results also showed that pancreatic cancer-derived exosomes increased MDSC (CD11b^+^GR1^+^cells) frequency in peripheral blood after exosome “education.”

We found that Panc02-H7 cell-derived exosomes induced liver pre-metastatic niche formation in naïve mice and consequently increased primary tumor growth and liver metastatic burden. We then identified exosomal proteins from Panc02 and Panc02-H7 cells via iTRAQ-based quantitative proteomic analysis. iTRAQ is currently one of the most robust methods of peptide labeling-based protein quantification. Our study identified more proteins than previous exosome proteomic studies [28–29], and nearly all of the 25 proteins most frequently identified in the ExoCarta databank as exosomal markers (http://exocarta.org/exosome_markers).

The profuse desmoplastic stroma forces pancreatic cancer cells to adapt their metabolisms to the hostile microenvironment. Metabolic reprogramming is essential for cancer cell survival and optimized growth in metastatic site microenvironments [30–31], and was recently recognized as a pancreatic cancer hallmark [32]. Cancer cell metabolic reprogramming may be a key pancreatic cancer progression and metastasis driver. Pathway analysis of differentially expressed proteins revealed that exosomal proteins are related to metabolism and cancer-related signaling pathways, including pyruvate metabolism, glutathione metabolism, glycolysis/gluconeogenesis, and alanine, aspartate, and glutamate metabolism.

In conclusion, our analyses demonstrated that metabolism-related signaling pathways were involved in exosome-mediated intracellular communication. We found that Panc02-H7-derived exosomes reduced Panc02 cell adhesion, and increased migration and invasion, enhancing the metastatic nature of these cells. In a mouse model, Panc02-H7 exosomes induced liver pre-metastatic niche formation and promoted primary tumor growth and liver metastasis. Further studies are needed to confirm whether the exosome-specific proteins identified in our bioinformatics studies are potential candidate pancreatic cancer diagnostic/prognostic markers or novel therapeutic targets.

## MATERIALS AND METHODS

### Chemicals and reagents

RPMI 1640, fetal bovine serum (FBS), proteases, proteases inhibitors, and antibiotics were purchased from Gibco-BRL (Shanghai, China). PKH67 membrane dye and 5-(N, N-Dimethyl) amiloride hydrochloride (DMA) were purchased from Sigma-Aldrich (MO, USA). Trans-well chambers were purchased from Corning Life Sciences (MA, USA). All iTRAQ reagents and buffers were purchased from Applied Biosystems, Inc. (Foster City, CA). All other reagents were of the highest analytical grades available and unless otherwise stated were purchased from Sigma-Aldrich (MO, USA).

### Cell lines and cultures

Corbett, et al. originally established the Panc02 murine PDAC cell line [33]. Wang, et al. established the Panc02-H7 sub-line using an *in vivo* selection method [11]. Panc02-H7cells are highly aggressive after implantation, with progressive growth in the pancreas, peritoneal dissemination, and distant metastasis to multiple organs, including the liver and lungs. All cell lines were gifted from Dr. Min Li (M.D. Anderson Cancer Center, USA). Cells were maintainedin RPMI 1640 supplemented with10% heat-inactivated FBS, 100 units/ml penicillin, 100 mg/ml streptomycin, and 0.25 mg/ml amphotericin B, and incubated at 37°C in humidified air with 5% CO_2_.

### Mice

Female C57BL/6 mice were purchased from Nanjing Chinchilla Technology Co. Ltd. (Nanjng, China) and used at 4–6 weeks of age. Animal experiments abided by the Guidelines for Animal Care and Use issued by the Southeast University Medical School Institutional Animal Care and Use Committee.

### Exosome isolation and purification

Panc02 and Panc02-H7cells were cultured to 70% confluence in 75 cm^2^ flasks in RPMI-1640medium supplemented with 10% exosome-free FBS, which had been depleted of bovine-derived exosomes by ultracentrifugation for 70 min at 100,000 g, followed by filtration through a 0.2-μm filter from Millipore (MA, USA). Supernatants collected from flasks were pelleted by centrifugation at 500 g for 10 min and 2,000 g for 20 min, resulting in floating cells and cell debris, respectively, and then further centrifuged at 20,000 g for 30 min to pellet larger microvesicles (MVs). Crude exosomes were prepared via ultracentrifugation of the supernatant at 100,000 g for 70 min, washing in PBS, and pelleting again by ultracentrifugation at 100,000 g for 70 min. Ultracentrifugation was always performed at 4°C. Crude exosome pellets were resuspended in 1ml PBS and then filtered (0.22 μm). Purified exosomes were obtained as previously described with minor modification [28]. Briefly, PBS-suspended exosome preparations were diluted in 3.5 ml PBS and layered on top of a density cushion composed of 20 mM Tris/30% sucrose/deuteriumoxide (D_2_O)/HCL pH 7.35 (0.5 ml) forming a visible interphase. Samples were ultracentrifuged at 100,000 g for 70 min. Exosomes contained in the 30% sucrose/D_2_O/Tris cushion and interphase were diluted five times with PBS and centrifugedat 120,000 g for 70 min. The final exosome pellets (Panc02 EXO and Panc02-H7 EXO) of higher purity were resuspended in PBS. We measured purified exosome total protein concentrations using the Bradford assay (Bio-Rad Laboratories, Hercules, CA), and purified exosome were stored at −80°C until use.

### Transmission electron microscopy (TEM)

Purified exosomes were fixed in 2% paraformaldehyde (w/v) in 200 mM phosphate buffer (pH 7.4). Fixed exosomes were dripped onto Formvar carbon-coated 200 mesh copper grids and absorbed at roomtemperature (RT) for 10 min. Excess liquid was removed with filter paper. Adsorbed exosomes were negatively stained with 3% phosphotungstic acid at RT for 5 min, dried with an incandescent lampfor 2 min, and observed viaTEM (JEM-2010; JEOL, Ltd., Tokyo, Japan) operating at 80.0 kV. Images were obtained using a cooled slow CCD camera.

### Western blotting

Exosome, cell, and liver tissue protein concentrations were measured with the Bradford assay. Proteins were separated by sodium dodecyl sulfate polyacrylamide gel electrophoresis (SDS-PAGE) and then transferred to polyvinylidine difluoride (PVDF) membranes. Membranes were incubated with primary antibodies at 4°C overnight in a buffer containing 5% skim milk, and then with a horseradish peroxidase (HRP)-conjugated secondary antibody at 37°C for 2 h. For exosome validation, primary antibodies against TSG101 (1:1000), CD9 (1:1000), MHC-1 (1:1000), and Cytochrome C (1:1000) were purchased from Abcam (Cambridge, UK). For cell migration and invasion assays, primary antibodies against CXCR4(1:500) and MMP-9 (1:500) were purchased from Boster (Wuhan, China). For liver pre-metastatic niche formation assays, primary antibodies against CD11b (1:1000), S100A8(1:1000), and S100A9 (1:1000) were purchased from Santa Cruz (CA, USA). For MS-identified candidates, primary antibodies against FRA2 (1:1000), IGF2BP2 (1:1000), and S100A11 (1:1000) were purchased from Abcam (Cambridge, UK). Selenium binding protein 1(SBP1, 1:500), CKAP4 (1:500), and aldolase C (ALDOC,1:500) were purchased from Bioss(Beijing, China). Protein-band densities were analyzed quantitatively using ImageJ software (NIH, USA).

### Exosome fluorescent labeling and uptake assay

Exosomes were stained following the manufacturer's instructions. Briefly, Panc02-H7 EXOswere labeled using the green lipophilic fluorescent dye, PKH67 (Sigma-Aldrich, St. Louis, MO) for 5 min. The reaction was terminated via addition of exosome-free FBS. To remove excess dye, PKH67-labeled exosomes were pelleted at 100,000 g for 70 min, washed three times with PBS, and resuspended in RPMI-1640 medium. Panc02 cells were inoculated in 6-well plates with exosome-free medium for 24 h. Cells were incubated with labeled exosomes for 5 h, washed three times with PBS, fixed with 4% paraformaldehyde for 15 min, and mounted with DAPI nuclear stain (1:100, Life Technologies, Grand Island, NY). Images were obtained using an inverted fluorescence microscope (Carl Zeiss, Germany).

### MTT cell adhesion, wound-healing, and invasion assays

For the cell adhesion assay, 96-well plates from Thermo Scientific (Shanghai China) were incubated at 37°C with Matrigel for 1 h and then terminated with PBS containing 1% BSA overnight. After exposure to PBS (control group),100μg/ml Panc02-H7 EXOs (EXO group), or 7 mmol/L amiloride (exosomes-depression, Exo-D group), for 24 h, Panc02 cells were suspended in serum-free medium. A Panc02 cell suspension (1×10_4_cells/100μl) was then added to each well and incubated at 37°C for 3 h. Plates were washed three times with PBS to remove unattached cells. Remaining Panc02 cells were reacted with MTT (5 mg/ml) for 4 h at 37°C, then dissolved in DMSO. The absorbance of each well was measured with ELX800 Absorbance Microplate Reader (Bio-TEK Co, Winooski, VT, USA) at 490 nm. OD values represent the number of adherent cells.

For the wound-healing assay, approximately 1×10^5^ Panc02 cells were inoculated in 6-well plates. After cultures reached 60% confluence, the monolayer was scratched using a 100 μl pipette tip. Attached cells were washed twice with PBS and incubated with medium as described for the cell adhesion assay. Each group (control, EXO, and Exo-D) was cultured in triplicate. Wound healing was analyzed under a microscope and images were captured at 0 and 24 h. The denuded area was measured viaImageJ (http://rsbweb.nih.gov). Cell motility was quantified using the formula:% of recovery = (A_t=0_-A_t=24_)/A_t=0_×100% (A_t=0_ is the denuded area measured immediately after wounding, A_t=24_ is the denuded area measured 24 h after incubation). After the experiment, cells were harvested and protein was extracted for Western-blot analysis.

For the Boyden chamber (invasion) assay, Panc02 cells were divided into three groups and pretreated for 24h as described adhesion assay. Cells (5×10^4^) were harvested and inoculated into upper transwell chambers in 5% FBS RPMI-1640 medium. 20% FBS RPMI-1640 medium was added into the lower chamber. Following 24h incubation, chamber upper surfaces were wiped with cotton swabs, and invading cells were fixed and stained with crystalviolet. Invading cells were counted in three randomly selected microscope fields for each transwell.

### Tissue distributions of pancreatic cancer-derived exosomes

Purified exosomes were labeled using PKH67 as previously described. PKH67-labeled exosomes from Panc02 and Panc02-H7 cells were injected intravenously into syngeneic C57B/L6 mice (20 μg exosomes/mouse). At 24 hpi, liver, lung, spleen, kidney, brain, and bone marrow tissues were harvested. Bone marrow cells were flushed from both the tibia and femurfor confocal microscopy. As controls, mice were injected with equivalent particle numbers of PKH67-labeled synthetic 100μm unilamellar liposomes. Fluorescence intensity was quantified using IPP6.0software (Media Cybernetics) to assess PKH67-labeled exosometissue distributions.

### Exosome-induced liver pre-metastatic niche formation assessment

Panc02-H7- and Panc02-derived exosomes (10μg each) were injected intravenously (tail vein) into C57B/L6 mice every other day. PBS was used as a control. At 12 d post-injection (dpi), livers were harvested for IF and Western blotting analysis. Peripheral blood obtained from mice was assessed using flow cytometry.

### Exosome-induced pancreatic cancer growth and liver metastasis assessment

We established a pancreatic-cancer SOI metastatic model with Panc02 cells in C57BL/6 mice as described in our previous study [34]. Panc02-H7 EXOs and Panc02 EXOs (10 μg) were injected intravenously (tail vein) into C57B/L6 mice three days per week, starting one week following SOI and continuing for three weeks. Mice were sacrificed 15 and 30 d after SOI. After anesthesia (isoflurane), mice were examined via laparotomy and thoracotomy. All solid organs were harvested and stained with hematoxylin and eosin (H&E) to assess metastases. Liver tissue was further assessed via IHC and Masson's trichrome staining to determine liver metastatic niche changes. Primary tumor volume was calculated using the formula: V = 0.5×a×b^2^, where a and b represent the long and short diameters of the tumor, respectively. PBS was used as a control.

### H&E staining, IHC, IF, and Masson's trichrome staining

Tumors and tissues were fixed in 10% neutral buffered formalin at least one d before paraffin embedding. Serial 4 μm sections were cut and stained with H&E for histopathological examination. Two pathologists independently counted the number of neutrophils in 10 high power fields.

IHC analysis was performed on formalin-fixed, paraffin-embedded tissue sections. Deparaffinization, antigen retrieval, and antigen-antibody reactions were performed using an automated DAKO Envision with Dual Link system-HRP. Tissue sections were incubated with primary antibodies followed by HRP-conjugated secondary antibody (from DAKO envision kit). Primary antibodies against fibronectin (1:100),α-SMA (1:50), S100A8 (1:50), S100A9 (1:50), and F4/80 (1:50) were purchased from Proteintech (Wuhan, China). Stain was developed using diamino-benzidine and counterstained with haematoxylin. IHC analyses were evaluated by two pathologists.

For IF, murine liver tissues were embedded in optimal cutting temperature (OCT) medium and stored at −80°C. Tissue sections were fixed in ice-cold acetone, permeabilized in PBS with 0.1% Triton X-100, blocked in PBS with 8% normal goat serum, and stained with primary antibodies to CD11b (1:500), P-STAT3 (1:500), CD45 (1:500), fibronectin (1:500), or α-SMA (1:500) (Santa Cruz). Tissue sections were then washed in PBS, stained with secondary antibodies conjugated to AlexaFluor 488 or AlexaFluor 594 (1:500, Life Technologies) and counter stained with DAPI (1:100, Life Technologies) to detect nuclei. Images were obtained with fluorescence microscopy (Olympus, BX43).

Masson's trichrome staining for connective tissue was performed according to the manufacturer's instructions (Abcam). IHC, IF, and Masson's trichrome staining images were processed and analyzed via IPP6.0 software (Media Cybernetics).

### Flow cytometry and antibodies

Peripheral blood was obtained by retro-orbital bleeding directly into EDTA anticoagulant tubes (Sarstedt, Newton, NC). Red blood cells were lysed using ACK lysis buffer (Gibco-BRL, Shanghai, China). Fc-receptors were blocked using anti-CD16/CD32 (BD Bioscience, Bedford, MA) before cell suspensions were incubated with fluorochrome-conjugated antibodies (mouse-CD11b-FITC and mouse-Ly-6G (Gr-1) PE, eBioscience, CA, USA) diluted in PBS with 1% BSA. Flow cytometry was performed using a Cytomics FC 500 flow cytometer (Beckman Coulter). Datawere analyzed using FlowJo software (TreeStar, Ashland, OR, USA).

### Protein preparation for quantitative proteomic analysis of pancreatic cancer-derived exosomes

Exosomes were suspended in lysis buffer (7 M urea, 2 M thiourea, 4%CHAPS, 40 mM Tris-HCl, pH 8.5,1mM PMSF, 2mM EDTA), and sonicated in ice. Proteins were reduced with 10 mM DTT (final concentration) at 56°C for 1 h and then alkylated with 55 mM IAM (final concentration) in the dark for 1 h. The reduced and alkylated protein mixtures were precipitated by adding 4×volume of chilled acetone at −20°C overnight. After centrifugation at 4°C and 30,000 g, the pellet was dissolved in 0.5 M TEAB (Applied Biosystems, Milan, Italy) and sonicated in ice. After centrifuguation at 30,000 g and 4°C, the supernatant protein concentration was measured with the Bradford assay. Proteins in the supernatant were stored at −80°C until use.

### iTRAQ labeling and strong cation exchange fractionation

**Figure 9:**
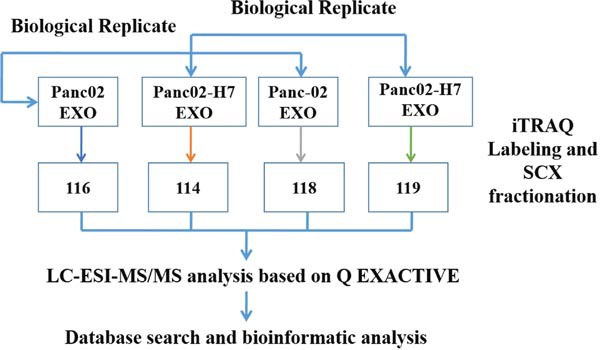
iTRAQ labeling experimental design schematic Panc02 and Panc02-H7 cell-derived exosomes (EXO) were labeled with iTRAQ tags, 116 and 114, respectively, and another pair of biological replicates of the same sample was labeled with iTRAQ tags, 118 and 119, respectively.

Protein (100μg) from each exosome sample was digested with Trypsin Gold (Promega, Madison, WI, USA) at a 30:1 protein:trypsin ratio, at 37°C for 16 h, and then dried by vacuum centrifugation. Peptides werereconstituted in 0.5 M TEAB and processed according to the manufacturer's protocol for 4-plex iTRAQ reagent. Briefly, one unit of iTRAQ reagent was thawed and reconstituted in 24 μl isopropanol. Samples were labeled with iTRAQ tags as follows: Panc02 EXO and Panc02-H7 EXO were labeled with iTRAQ tags, 116 and 114, respectively, and biological replicates of the same exosomes were labeled with iTRAQ tags, 118 and 119, respectively. Peptides were labeled with the isobaric tags, incubated at room temperature for 2h, and then pooled and dried by vacuum centrifugation. Strong cation exchange (SCX) chromatography was performed with a LC-20AB HPLC pump system (Shimadzu, Kyoto, Japan). iTRAQ-labeled peptide mixtures were reconstituted with 4 ml buffer A (25 mM NaH_2_PO_4_ in 25% ACN, pH 2.7) and loaded onto a 4.6×250 mm Ultremex SCX column containing 5 μm particles. Peptides were eluted at a flow rate of 1 ml/min with a gradient of buffer A for 10 min, 5–60% buffer B (25mM NaH_2_PO_4_, 1 M KCl in 25% ACN, pH 2.7) for 27 min, and 60–100% buffer B for 1 min. The system was then maintained at 100% buffer B for 1 min before equilibrating with buffer A for 10 min prior to the next injection. Elution was monitored by measuring the absorbance at 214 nm, and fractions were collected every 1 min. Eluted peptides were pooled into 20 fractions, de-salted with a Strata X C18 column (Phenomenex), and vacuum-dried. The iTRAQ workflow is shown in (Figure 9).

### LC-ESI-MS/MS analysis based on Q EXACTIVE

Each fraction was resuspended in buffer A (2% ACN, 0.1%FA) and centrifuged at 20,000 g for 10 min. The average final peptide concentration was approximately 0.5 μg/μl. 10 μl supernatant was loaded on an LC-20AD nanoHPLC (Shimadzu, Kyoto, Japan) with the autosampler onto a 2-cm C18 trap column. Peptides were eluted onto a 10-cm analytical C18 column packed in-house. Samples were loaded at 8 μl/min for 4 min, and then the 44-min gradient was run at 300 nl/min from 2–35% B (98%ACN, 0.1%FA) followed by a 2-min linear gradient to 80% B, maintenance at 80% B for 4 min, and a return to 5% over 1 min. Peptides were subjected to nano-electrospray ionization followed by tandem mass spectrometry (MS/MS) in a QEXACTIVE (Thermo Fisher Scientific, San Jose, CA) coupled online to the HPLC. Intact peptides were detected in the Orbitrap at a resolution of 70,000. Peptides were selected for MS/MS using the high-energy collision dissociation (HCD) operating mode with a normalized collision energy setting of 27.0. Ion fragments were detected in the Orbitrap at a resolution of 17,500. A data-dependent procedure that alternated between one MS scan followed by 15 MS/MS scans was applied for the 15 most abundant precursor ions above a threshold ion count of 20,000 in the MS survey scan, with a following Dynamic Exclusion duration of 15 s. The electrospray voltage applied was 1.6 kV. Automatic gain control (AGC) was used to optimize the spectra generated by the Orbitrap. The AGC target for full MS was 3e6, and 1e5 for MS2. The m/z scan ranges were 350–2,000 Da for MS scans, and 100–1800Da for MS2 scans.

### MS data analysis

Raw data files acquired from the Orbitrap were converted into MGF files using Proteome Discoverer 1.2 (PD 1.2, Thermo) (5600 ms converter) and the MGF files were searched. Protein identification was performed using the Mascot search engine (Matrix Science, London, UK;version 2.3.02) against uniprot+exosome (mouse) databases containing 78,156 sequences. For protein identification, a mass tolerance of 10 ppm was permitted for intact peptide masses and 0.05 Da for fragmented ions, with allowance for one missed cleavage in the trypsin digest. Gln->pyro-Glu (N-term Q), oxidation (M), and Deamidated (NQ) were the potential variable modifications, and Carbamidomethyl (C), iTRAQ 4 plex (N-term), and iTRAQ 4 plex (K) were fixed modifications. Peptide charge states were set to +2 and +3. An automatic decoy database search was performed in Mascot by choosing the decoy checkbox, in which a random database sequence is generated and tested for raw spectra as well as the real database. To reduce the probability of false peptide identification, only peptides at the 95% confidence interval as determined by a Mascot probability analysis greater than “identity” were counted as identified. Each protein identification involved at least one unique peptide. Protein quantitation required that a given protein contain at least two unique spectra. Quantitative protein ratios were weighted and normalized by the median ratio in Mascot. We only used ratios with p<0.05, and only fold changes of >1.5 were considered significant.

### Bioinformatics analyses

Protein functional annotations were conducted using Blast2GO against the non-redundant protein database (NR;NCBI). The KEGG database (http://www.genome.jp/kegg/) was used to classify and group these identified proteins. Gene Ontology (GO) is an international standardization of gene function classification system. It provides a dynamically-updated, controlled vocabulary to describe gene and gene product attributes in the organism. GO ontologies describe molecular function, cellular component, and biological process. KEGG PATHWAY is a collection of manually drawn pathway maps representing molecular interaction and reaction networks.

### Statistical analysis

Quantitative data are presented as means±standard error of the mean (s.e.m.). Statistical analyses were performed using SPSS software, version 18.0. Data were compared using Student's *t*-test. P<0.05 was considered significant. For functional enrichment analysis using DAVID, cluster with an enrichment score >1.3 (−log(p-value)) were considered significant (the geometric mean of the p-values in a significant cluster was <0.05).

## SUPPLEMENTARY MATERIALS TABLE




