# Supplementary material for: Pancreatic cancer-derived exosomes promote tumor metastasis and liver pre-metastatic niche formation
Source: Oncotarget. 2017 Jun 28;8(38):63461–83. doi: 10.18632/oncotarget.18831 (PMC5609937; doi:10.18632/oncotarget.18831)
Supplement: Supplementary file 1 [file oncotarget-08-63461-s001.pdf]

Pancreatic cancer-derived exosomes promote tumor metastasis and liver pre-metastatic niche formation

SUPPLEMENTARY TABLE

Supplementary Table 1: All proteins were identified via iTRAQ-based proteomic analysis in the two types of pancreatic cancer exosomes

See Supplementary File 1
